# The Gut–Heart Axis: A Microbiome-Centered Perspective on Heart Failure

**DOI:** 10.3390/ijms27146163

**Published:** 2026-07-10

**Authors:** Diana-Elena David, Tatiana Dramba, Stefan Andrei Chiriac, Cringuta Mariana Paraschiv, Gabriela Grigorasi, Alexandru Gabriel David, Vasile Valeriu Lupu, Ancuta Lupu, Gabriela Păduraru, Leonard Iosif Pertea, Oana-Raluca Temneanu, Irina Mihaela Esanu

**Affiliations:** 1 Grigore T. Popa University of Medicine and Pharmacy, 700115 Iasi, Romania; diana-elena.david@d.umfiasi.ro (D.-E.D.); andrei-chiriac@umfiasi.ro (S.A.C.); cringuta.paraschiv@umfiasi.ro (C.M.P.); gabriela.tiulica@yahoo.com (G.G.); irina.esanu@umfiasi.ro (I.M.E.); 2 St. Mary Emergency Children Hospital, 700309 Iasi, Romania; alex.david25@yahoo.com

**Keywords:** gut microbiota, cardiovascular disease, dysbiosis, TMAO, SCFAs, gut–heart axis, gut microbiota, heart failure

## Abstract

In recent years, gut microbiota has emerged as a central modulator of cardiovascular health and disease. This has led to a transition from the old understanding of cardiovascular pathology as a largely cardiac-centric problem to a systemic, multi-organ process. A growing body of evidence demonstrates that changes in the makeup of gut microbes, generally called dysbiosis, are significant in the development and progression of cardiovascular illnesses, including heart failure. Moreover, there are bidirectional interactions between the failing heart and the gut. In heart failure, impaired hemodynamics and venous congestion further worsen intestinal hypoperfusion and barrier dysfunction in a self-perpetuating cycle that exacerbates dysbiosis and systemic inflammation. The gut–heart axis offers a fresh paradigm for illness progression beyond classical neurohormonal and hemodynamic processes. The gut microbiota acts as an endocrine organ by producing bioactive metabolites such as TMAO (trimethylamine N-oxide), SCFA (short-chain fatty acids) and bile acids, which, via several routes, have a serious impact on host health and disease. This narrative review aims to summarize the current evidence for the gut microbiota as a new cardiovascular risk factor, focusing on biological mechanisms and clinical and epidemiological evidence.

## 1. Introduction

### 1.1. Cardiovascular Diseases—A Global Burden

Cardiovascular diseases (CVDs) nevertheless continue to be the number one cause of morbidity and mortality worldwide, representing a huge health burden and a large percentage of premature deaths [1]. Despite improvements in prevention and control, traditional risk factors such as hypertension, dyslipidemia, diabetes mellitus, smoking, and obesity cannot explain the totality of the residual cardiovascular risk in many populations [2]. This has led to increased interest in the search for novel possibly modifiable factors impacting cardiovascular pathophysiology.

In recent years, the gut microbiome has become a central regulator of host homeostasis and increasing evidence has been linked to cardiovascular health and illness [2,3]. The gut microbiome is a complex and dynamic ecosystem of microorganisms that play a key role in physiological functions such as nutrition, metabolism, immune system modulation and intestinal barrier integrity. Changes in microbial composition and activity (dysbiosis) have been associated with multiple cardiometabolic diseases, including atherosclerosis, hypertension, and heart failure [3,4].

Hypertension is one of the major risk factors of cardio-cerebral vascular diseases, which may lead to dysbiosis of gut microbiota and dysfunction of the gut barrier. This is driven by an increase in hydrogen sulfide, lipopolysaccharide (LPS) and pathogenic bacteria and the reduction in short-chain fatty acid (SCFA) generating bacterial populations. These changes increase intestinal permeability and the breakdown of tight junction proteins. In addition, decreased alpha diversity and increased abundance of LPS-producing Gram-negative bacteria trigger pro-inflammatory responses, resulting in dysregulated blood pressure and hypertension.

The gut microbiome is thought to affect the evolution and course of heart failure (HF) and is considered a prognostic factor in heart failure with preserved ejection fraction (HFpEF) and heart failure with reduced ejection fraction (HFrEF) [5,6].

A key biological connection between the gut microbiota and cardiovascular disease (CVD) involves the production of metabolites derived from food substrates. Trimethylamine N-oxide (TMAO) has been extensively studied. TMAO is formed through a pathway in which gut bacteria catabolize dietary nutrients (e.g., choline and L-carnitine) to trimethylamine, which is then oxidized in the liver. Increased plasma levels of TMAO have been implicated in the increased risk of major adverse cardiovascular events and mortality, possibly through mechanisms of increased atherosclerosis, altered cholesterol metabolism and increased platelet reactivity [7,8].

Conversely, certain metabolites from microbiota, such as short-chain fatty acids (SCFAs), can have preventive cardiovascular effects through modulation of inflammation, endothelial function, and metabolic homeostasis [7].

The concept of a bidirectional “gut–heart axis,” reflecting the complex connection between gut bacteria and heart function, is attracting increasing interest [4]. Beyond metabolite-mediated effects, increased intestinal permeability and the resulting transfer of microbial components, such as lipopolysaccharides, may contribute to systemic inflammation and promote the course of cardiovascular diseases. While there is increasing evidence of research supporting these associations, significant challenges remain, including diversity in study designs and difficulty establishing causality.

### 1.2. Overview of the Intestinal Microbiome Architecture

The gut microbiome is a complex and dynamic ecosystem containing trillions of species, including bacteria, archaea, viruses, and fungi, predominantly found in the gastrointestinal tract [9,10]. This population of microorganisms functions as a metabolically active “organ” and plays a major role in a variety of host physiological activities, such as digestion, the absorption of nutrition, synthesis of bioactive substances and the regulation of immunological homeostasis [10,11]. Recent developments in high-throughput sequencing technology and metagenomic analysis have significantly improved our understanding of the taxonomical composition and functional potential of the gut microbiome [12,13].

**Figure 1:**
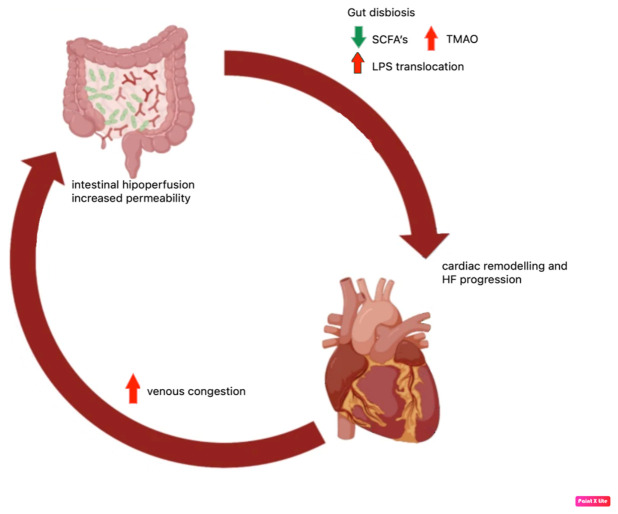
Proposed vicious cycle linking heart failure and gut dysbiosis—reduced cardiac output and venous congestion promote intestinal hypoperfusion and barrier dysfunction, leading to microbial translocation, systemic inflammation, and adverse cardiac remodeling. Created by the authors.

The gut microbiota of healthy persons is predominantly composed of the bacterial phyla Firmicutes and Bacteroidetes, with smaller proportions of Actinobacteria, Proteobacteria and Verrucomicrobia [14]. The relative richness and diversity of these microbial communities are influenced by a number of host-related and environmental factors such as genetics, age, diet, medication usage (particularly antibiotics), and lifestyle practices (Figure 1). Diet is a known major determinant of the composition of the gut microbiome, with long-term dietary patterns contributing to defining the microbial enterotypes and metabolic consequences [15,16]. (Figure 1).

One of the key features of a healthy gut microbiota is its diversity and functional strength. Dysbiosis, which is a decreased microbial diversity and changed community structure, has been associated with several clinical diseases, including metabolic, inflammatory and cardiovascular diseases [17,18]. Dysbiosis can involve the loss of beneficial microbial taxa, the overgrowth of potentially harmful bacteria, and changes in microbial metabolic pathways, ultimately leading to reduced gut barrier function and systemic effects on human physiology [19].

The main effect of the gut microbiome on host–microbe interactions is via the production of metabolites. They include short-chain fatty acids (SCFAs), bile acid derivatives, and several signaling molecules that affect energy metabolism, immune responses and inflammatory pathways [20,21]. Thus, the composition and metabolic functions of the gut microbiome are being highly acknowledged as key contributors in health and disease, especially in relation to cardiovascular risk.

The intestinal mucosal surface represents the interface of the gut microbiota and the host, and it has multiple functions to preserve the integrity of the intestinal epithelial barrier. Bacterial endotoxins and bacteria and their metabolites may cross the intestinal barrier and enter the blood, which may cause autoimmune disorders. Several cardiovascular disorders, including atherosclerosis, myocardial infarction, cardiomyopathies and heart failure, rely on immune dysregulation and inflammation [22,23].

As this article is a narrative review, no formal systematic review protocol was applied. A literature search was conducted using PubMed, ScienceDirect and CrossRef databases. The search covered publications from January 2012 to January 2026. The following keywords were used in various combinations: “gut microbiota” OR “intestinal microbiota” OR “gut dysbiosis” AND “cardiovascular disease” OR “heart failure” OR “hypertension” AND “trimethylamine N-oxide” OR “TMAO” OR “short-chain fatty acids” OR “SCFAs” OR “gut–heart axis”. Articles were considered eligible if they were published in peer-reviewed journals and provided mechanistic, experimental, translational, or clinical evidence regarding interactions between the gut microbiota and cardiovascular diseases. Original research articles, systematic reviews, meta-analyses, and relevant landmark studies were included.

## 2. Changes in the Gut Microbiota of HF Patients

Gut dysbiosis has been associated with heart failure through multiple molecular mechanisms, such as compromised gut barrier function, bacterial translocation and endotoxemia, which may induce systemic inflammation and detrimental cardiac remodeling. The main biomarkers discovered as markers of intestinal permeability and low-grade inflammation in patients with heart failure are zonulin and lipopolysaccharides (LPS) [24,25].

### 2.1. Evidence from Systematic Reviews and Meta-Analyses: Consistent Dysbiosis Patterns

Systematic reviews and meta-analyses give the strongest evidence of gut microbiota alterations in cardiovascular disease and HF. Martins et al. [26] reported a consistent pattern in cardiovascular populations with a decrease in the prevalence of SCFA-producing bacteria and an enrichment of pro-inflammatory taxa. Moreover, despite the differences in methodologies of the research, the microbial composition remains similar, with an increase in Streptococcus and Proteobacteria and a decrease in Faecalibacterium.

Similarly, Huang et al. [27] confirmed lower alpha diversity and large-scale alterations in beta diversity as the hallmarks of HF by meta-analysis of 16S rRNA sequencing studies, which corroborates the concept of microbial ecological instability in HF patients. Furthermore, although many studies differed in the precise taxa identified, the directionality of changes was mainly consistent across cohorts.

These findings are further reinforced by Anderson et al. [28], who specifically measured gut microbiota and trimethylamine N-oxide (TMAO) in HF and established a similar relationship between dysbiosis, decreased SCFA-producing bacteria, and increased circulating TMAO levels. However, the authors also reported considerable variation in effect magnitude between trials, suggesting that microbiota–metabolite interactions are influenced by confounding factors such as nutrition, renal function and illness severity.

### 2.2. Observational Cohort Studies: HF Phenotype-Specific Microbial Signatures

Further clinical investigations explain these results by identification of HF phenotype-specific changes in microorganisms. In HF with preserved ejection fraction (HFpEF) patients, Beale et al. [29] found substantial differences in both alpha and beta diversity compared to controls, but without a consistent alteration in the Firmicutes/Bacteroidetes ratio. Moreover, this study showed a selective depletion of SCFA-producing bacteria, particularly Ruminococcus, after adjustment for diet, BMI and comorbidities.

Larger compositional changes, however, have been reported in studies in severe chronic HF populations. Sun et al. [30] detected a decline in microbial diversity with dominance of Pseudomonadota and loss of beneficial taxa. Kummen et al. [31] confirmed these observations, showing decreased levels of key SCFA-producing species, especially from the Lachnospiraceae family, in two different cohorts. This study provides evidence that SCFA-producing bacteria depletion is a major component of HF-linked dysbiosis in independent populations.

### 2.3. HF with Reduced Ejection Fraction: Loss of Core Microbiota and Inflammatory Shift

In HFrEF, Luedde et al. [32] found a considerable loss of the core intestinal microbiota, including Ruminococcaceae, Blautia, and Collinsella, and a reduction in microbial diversity. These results support the hypothesis that HF is characterized not only by proliferation of potentially harmful bacteria but also the collapse of beneficial microbial networks.

Also, Gram-negative organisms such as Escherichia-Shigella, Klebsiella, and Enterococcus were enriched in Sun et al. [30] and other cohorts. This switch to lipopolysaccharide (LPS) generating bacteria provides a molecular link to systemic inflammation, since enhanced microbial translocation could stimulate immune activation and accelerate HF progression. These findings were later confirmed by other systematic reviews and meta-analyses carried out by Anderson et al. and Huang et al., both of which found decreased microbial richness to be a defining feature of heart failure populations. The consistency of this discovery across multiple heart failure phenotypes and independent cohorts shows that diminished ecological stability of the gut microbiota may be a common feature of chronic heart failure rather than a disease subtype-specific phenomenon [27,28].

### 2.4. TMAO and Gut-Derived Metabolites: Association vs. Causality

Among the metabolites of gut microbiota, trimethylamine N-oxide (TMAO) has been researched most extensively. Most observational studies and meta-analyses have shown that high circulating levels of TMAO are related to worse HF outcomes, including mortality, hospitalization rates and disease severity [28,33].

In a pivotal study, Wang et al. (2011) [34] provided the first mechanistic evidence that gut microbiota-dependent metabolism contributes to cardiovascular disease. Using data from human cohorts and experimental animal models, they found an association between elevated TMAO levels and increased development of atherosclerotic lesions and that reduction in gut microbiota with antibiotics significantly decreased TMAO production and slowed disease progression. Mechanistically, the study found that there was a direct causal connection between gut microbial metabolism, host hepatic processing and cardiovascular disease. This study paved the way for understanding that gut microbiota-derived metabolites are not just indicators but active participants in the causes of cardiovascular disease. These observations provide a critical conceptual foundation to evaluate subsequent studies relating increased circulating TMAO levels to unfavorable outcomes and disease severity in the setting of heart failure [34].

Israr et al. [35] further showed that greater levels of gut-derived metabolites, particularly TMAO, are associated with worse outcomes in acute HF beyond recognized prognostic indicators. Likewise, Jarmukhanov et al. [33] reviewed the molecular pathways that TMAO could induce endothelial dysfunction, oxidative stress, fibrosis and ventricular remodeling.

But causality is not certain, despite the overwhelming association evidence. The persistence of heterogeneity among investigations [28] implies that TMAO may work as a mediator and a marker of larger metabolic and microbial dysfunction, rather than a standalone pathogenic cause.

### 2.5. SCFA Depletion: A Convergent Mechanistic Pathway Across Studies

The decrease in SCFA-producing bacteria is one of the most consistent findings; however, taxonomy data are variable among research. The reduction in taxa such as Ruminococcus, Faecalibacterium and Lachnospiraceae is consistent with Beale et al. [29], Kummen et al. [31], Luedde et al. [32] and several meta-analyses [27,28]. This convergence shows that the functional loss of SCFA production may be more biologically meaningful than taxonomic distinctions between individual samples. SCFAs have been shown to preserve the integrity of the gut barrier, modulate immunological responses and exert cardioprotective effects, and hence their depletion may lead to increased intestinal permeability and systemic inflammation in HF. (Summary in Table 1).

### 2.6. Confounding Factors Influencing Gut Microbiota Alterations in Heart Failure

Interpretation of gut microbiome changes in heart failure is hampered by the large effect of several confounding variables that independently affect microbial composition and metabolite production. Some of the disparities found between patients with heart failure and healthy controls may be explained partially or totally by these factors.

Diet is one of the most powerful modulators of gut microbiota composition, and especially fiber consumption, which directly influences the amount of short-chain fatty acid (SCFA)-producing bacteria. Reduced dietary fiber consumption, as often observed in patients with severe heart failure, may thus contribute independently to the depletion of beneficial microbial taxa reported across studies.

Another key confounding factor is pharmacological therapy. Commonly used cardiovascular and metabolic medicines like antibiotics, proton pump inhibitors, metformin, statins, SGLT2 inhibitors and diuretics have all been proven to change gut bacteria diversity and composition. For instance, diuretics and antibiotics could significantly affect gut permeability and microbial homeostasis, and so might mimic or worsen disease-associated dysbiosis [40,41,42].

Interpretation is further complicated by comorbidities including chronic kidney disease, diabetes mellitus and obesity, each independently associated with characteristic microbiome signatures and an altered metabolite profile including increased circulating TMAO levels and decreased SCFA production. Demographic and clinical characteristics such as age, sex, geographic region, hospitalization status, and severity of heart failure (HFrEF versus HFpEF) all contribute to variation across studies. For example, hospitalized patients with advanced disease are more likely to have severe dysbiosis, which may not be representative of the stable outpatient population [43,44].

All combined, these confounding factors underline the difficulty in attributing microbiome abnormalities to the pathogenesis of heart failure in particular. Significant indications of reduced microbial diversity and altered metabolite profiles are found, but future investigations need to account for these factors to differentiate disease-specific microbial signatures from secondary or treatment-related effects.

## 3. Heart–Gut Axis Interplay

Recent evidence emphasizes the importance of the gut–heart axis in the pathophysiology of heart failure. Florek et al. (2025) [45] present a review of the gut microbiota in the origin of disease and highlight the function of intestinal dysbiosis in systemic inflammation and cardiac remodeling. Heart failure is related to intestinal hypoperfusion and venous congestion compromising the integrity of the epithelial barrier and facilitating the entry into the circulation of microbial products such as lipopolysaccharides. This results in a higher degree of cardiac dysfunction through the release of pro-inflammatory cytokines and activation of innate immunity pathways. In addition, metabolites of the microbiota are key regulators of cardiovascular homeostasis. Excessive trimethylamine N-oxide is linked to endothelial dysfunction and poor prognosis, in contrast to reduced synthesis of short-chain fatty acids, which leads to decreased anti-inflammatory protection. The gut microbiota and heart failure have a bidirectional link, with cardiac dysfunction altering microbial composition and establishing a self-perpetuating pathogenic cycle [45].

The bidirectionality of this relationship is also mentioned by Epelde, who describes the effect of changes in the microbiota on inflammation and metabolic pathways, but also on endothelial function, fluid balance and sodium absorption, all of which are central to the pathophysiology of heart failure [46]. This compositional change is consistent with mechanistic models demonstrating that a decreased SCFA level compromises both intestinal barrier integrity and anti-inflammatory signaling. Also, elevated TMAO levels are associated with endothelial dysfunction and poor cardiac remodeling, possibly through modulation of inflammatory and oxidative stress pathways [47].

There are differences in focus and interpretation with these converging results. Florek et al. underscore the variability and complexity of the impact of metabolites in different HF etiologies and NYHA stages. Epelde emphasizes widespread metabolic and hormonal perturbations beyond classic inflammatory paradigms.

The gut microbiome dysbiosis and heart failure are suggested to have a bidirectional association, including clinical and observational data. However, clear evidence from animal models suggests that cardiac failure alone might induce and perpetuate intestinal abnormalities, promoting systemic inflammatory pathways and potentially exacerbating disease progression. Experimental evidence strongly shows that heart failure directly causes disruption of the intestinal barrier. Cardiac failure generated profound disturbance of gut homeostasis, which included reduced intestinal barrier integrity, changed microbial composition and increased systemic endotoxin levels in a mouse pressure-overload model induced by transverse aortic constriction (TAC). TAC mice had more circulating lipopolysaccharide (LPS) and pro-inflammatory cytokines and lower expression of anti-inflammatory mediators in the colon. These modifications were accompanied by changes in the dominant bacterial phyla: Firmicutes, Proteobacteria and Actinobacteria, which indicate that cardiac dysfunction alone is capable of inducing dysbiosis in the gut. The importance of this model is that it provides mechanistic evidence that gut abnormalities are not only secondary connections but an active component of heart failure development through systemic inflammatory signaling pathways [48].

Further investigations suggest that the exact contribution of microbiota changes to HF progression may differ according to the HF phenotype. In particular, in the case of HF with preserved ejection fraction (HFpEF), inflammation and endothelial dysfunction mediated by the microbiome are emerging as central fields of investigation [49].

The role of the gut microbiota in heart failure can depend on the specific phenotype of heart failure. In their fundamental theory, Paulus and Tschöpe hypothesized that HFpEF develops due to a systemic pro-inflammatory state that is induced by frequent co-morbidities such as obesity, diabetes mellitus, hypertension, and chronic kidney disease. In this paradigm, persistent inflammation leads to coronary microvascular endothelial dysfunction, with impairment of nitric oxide bioavailability, cyclic guanosine monophosphate–protein kinase G signaling, cardiomyocyte stiffness, and myocardial fibrosis. This paradigm is of particular relevance for the gut–heart axis, where intestinal dysbiosis and enhanced gut permeability can further contribute to systemic inflammation due to microbial product translocation and synthesis of pro-inflammatory chemicals. Microbiota-derived mechanisms may therefore be more relevant in HFpEF, where inflammation and endothelial dysfunction are believed to be primary pathogenic factors [44].

This is further supported by Obokata et al., who extended the definition of HFpEF beyond simple diastolic dysfunction to a complex multisystem illness characterized by the interplay of cardiac, vascular, pulmonary, skeletal muscle, and metabolic derangements. They observed the considerable variations in HFpEF and emphasized the value of systemic inflammation, endothelial dysfunction, decreased reserve capacity and metabolic abnormalities in the course of the disease. The results suggest multiple mechanisms by which microbial dysbiosis can affect HFpEF via the gut–heart axis, including regulation of inflammatory signaling, endothelial function, oxidative stress, and metabolic balance. Conversely, gut problems in heart failure with reduced ejection fraction (HFrEF) seem to be more linked to hemodynamic changes such as lower cardiac output, intestinal hypoperfusion and venous congestion, which compromise intestinal barrier integrity and enhance endotoxemia. Together, these results support the hypothesis that gut microbiome modifications may be involved in both HF symptoms, although the processes and their proportional contribution are likely to be different between HFpEF and HFrEF [50].

The extent to which dysbiosis is a cause or a result of hemodynamic impairment and medication in heart failure is still contested. This calls for longitudinal and interventional investigations to elucidate these connections. Together, these recent studies support that targeting gut microbiota and its metabolites is a promising but still evolving theory in heart failure understanding.

### 3.1. Trimethylamine-N-Oxide

TMAO is consistently considered a factor for CVD and is generated from dietary sources including choline, betaine, phosphatidylcholine, lecithin, and L-carnitine. Choline, phosphatidylcholine and trimethylamine carnitine are often found in meat, egg yolks and high-fat dairy foods. (A) They are metabolized by intestinal bacteria to TMA, which is absorbed and transported to the liver via the portal circulation. These chemicals are transformed in a two-step process: TMA has been detected in 36 species and 102 genomes. Firmicutes, Proteobacteria and Actinobacteria produce TMA, whereas Bacteroidetes do not. Firmicutes (Anaerococcus, Clostridium, Desulfitobacterium, Enterococcus and Streptococcus) and Proteobacteria (Pseudomonas, Enterobacter, Proteus, Escherichia, Desulfovibrio, Actinobacter, Citrobacter and Klebsiella) have been related to TMA production [51]. (B) The microbiome-derived TMA molecule enters the host’s circulation and reaches hepatocytes where it is converted to TMAO by the flavin-containing monooxygenase (FMO) enzyme produced by the FMO gene in the liver, kidney, and other tissues [39,52,53].

New research mentions trimethylamine N-oxide (TMAO), produced by bacteria in the gut, as a significant factor in the development and progression of cardiovascular disease, causing complications such as heart failure. Higher circulating TMAO levels have been associated with worse clinical outcomes in heart failure, including higher risk of hospitalization and death in observational and mechanistic studies independent of traditional risk factors. In a meta-analysis of several cohorts, greater concentrations of TMAO in heart failure patients were related with an increased risk of major adverse cardiovascular events and all-cause mortality, suggesting the prognostic importance of TMAO in this population [54]. TMAO is considered as a factor that worsens the adverse cardiovascular remodeling by affecting endothelial dysfunction, inflammation, and oxidative stress, hence promoting the processes relevant to the progression of heart failure; however, clinical evidence is largely observational and does not confirm a direct causal role [55,56].

Recent large community-based cohort studies have shown that increased TMAO and associated metabolites were independently associated with increased incidence of heart failure, supporting the idea that gut microbiota-driven metabolite pathways influence disease progression before clinical manifestation [57].

These findings suggest TMAO as a potential predictive biomarker and a possible regulator of the heart–gut axis, linking microbial metabolism to cardiac failure.

### 3.2. Short-Chain Fatty Acids (SCFAs)

SCFAs are microbial metabolites produced during the fermentation of complex carbohydrates. These substances modify the composition of the microbiota and the motility of the gastrointestinal tract and, consequently, influence many host functions. The major short chain fatty acids are acetate, propionate and butyrate. Phylum Bacteroidetes produce acetate and butyrate, while Phylum Siliques exclusively produce butyrate. Short-chain fatty acids are positively associated with Alistipes putredinis, Bacteroides spp., Roseburia, Eubacterium rectale, and Faecal prausnitzii [58]. SCFAs also help maintain the integrity of the intestinal barrier by modulating the synthesis of tight junction proteins.

SCFAs, especially butyrate, modulate the intestinal barrier function, decreasing the permeability of the epithelium by increasing the expression of the tight junction proteins, zonulin and occludin. However, other studies show that high levels of butyrate are significant since they can negatively stimulate apoptosis of intestinal epithelial cells. This leads to endotoxemia through an influence on intestinal membrane permeability and consequent inflammation in subjects with a reduced abundance of butyrate-producing phyla [59].

Short-chain fatty acids can reduce blood lipid levels by blocking the synthesis of cholesterol and promoting its transit to the liver. Therefore, they are considered as a preventive factor in the progression of cardiovascular diseases [1].

Short-chain fatty acids (SCFAs) activate G-protein-coupled receptors (GPR41, GPR43 and GPR109A), which modulate the immune response by increasing anti-inflammatory cytokines (IL-10) and reducing systemic inflammation, a characteristic of heart failure (HF). They also inhibit histone deacetylases (HDACs), which controls gene expression to boost anti-inflammatory gene expression, improve gut barrier integrity, and stop bacterial endotoxin translocation, all of which are linked to heart failure (HF) [60]. Therefore, SCFAs maintain the integrity of the gut epithelium and reduce microbial translocation, hence lowering systemic inflammation and improving endothelial function [61].

### 3.3. Bile Acids

Beyond their role in the breakdown of lipids, bile acids (BAs) are becoming appreciated as essential signaling molecules regulating cardiovascular regulation. The field of bile acid physiology has evolved more rapidly with the development of bile acid responsive receptors, particularly the farnesoid X receptor (FXR) and the G protein-coupled bile acid receptor 1 (TGR5). Changes in bile acid metabolism are connected to cardiovascular illness, including heart failure, as suggested by new research. In a cross-sectional study, patients with chronic heart failure had an increased ratio of secondary to primary bile acids in the circulation, which was significantly associated with shorter overall survival in univariate analysis [62]. FXR activation has cardioprotective effects via the regulation of bile acid homeostasis and inhibition of NF-κB signaling, which reduces inflammation and improves myocardial function. Activation of TGR5 has been shown to have cytoprotective effects in cardiac tissue, enhancing myocardial adaptation to physiological, inotropic and hemodynamic stress in experimental conditions. These findings point out the dual role of bile acids as metabolic regulators and signaling molecules in cardiovascular pathology, suggesting that targeting FXR and TGR5 pathways would be a viable therapeutic approach for heart failure therapy [63,64].

While microbiota-derived metabolites such as TMAO, short-chain fatty acids and bile acids are often cited as mechanistic mediators in heart failure, we want to highlight that a considerable part of the supporting information comes from observational and cohort research. These study designs are fundamentally limited to differentiate causal correlations from associations driven by disease severity, renal dysfunction, nutritional consumption, comorbidities, and pharmacologic therapies. Thus, high concentrations of TMAO and other gut-derived metabolites should be viewed more as indicators of altered host–microbiome interactions rather than as proven direct drivers of heart failure progression. Experimental investigations have demonstrated biological evidence for mechanistic involvement, particularly through pathways involving inflammation, endothelial dysfunction and cardiac remodeling, although these have not been conclusively verified in interventional human trials.

To date, evidence favors a concept in which microbiota-derived metabolites are components of a complex bidirectional gut–heart axis that may play both mechanistic and reflective (biomarker) functions, rather than being definitive causal agents.

## 4. Heterogeneity of Gut Microbiome Findings in Heart Failure

Many studies have described changes in the makeup of gut microbiota in heart failure, although not all described taxonomic shifts have been similarly consistent across cohorts. There is a fairly consistent core set of findings across independent research, specifically the decrease in microbial diversity and loss of bacteria producing short-chain fatty acids (SCFAs), such as Faecalibacterium, Ruminococcus, Roseburia and members of the Lachnospiraceae family consistently found in both HFpEF and HFrEF populations, suggesting that loss of SCFA-associated microbial capabilities may constitute a common feature of heart failure-associated dysbiosis.

In contrast, a few of taxa-level changes demonstrate more cohort dependence and variability. For instance, many studies have shown enrichment of Proteobacteria, Escherichia-Shigella, Klebsiella, Enterococcus, Streptococcus, and Alistipes, but the extent and consistency of these alterations differ substantially amongst groups. Phylum-level ratios such as Firmicutes/Bacteroidetes (or Bacillota/Bacteroidota) led to conflicting results between studies, limiting their value as accurate markers of dysbiosis associated with heart failure.

One developing key idea is that functional changes in metabolism may be more repeatable than individual taxonomic changes. In this context, it has been suggested that disturbances in microbial amino acid metabolism may be possible factors in the pathophysiology of heart failure, recommending that future investigations should focus not only on microbial composition but also on microbial metabolic activity. Tuerhongjian et al. reviewed the interplay between gut microbiota and amino acid metabolism in heart failure and highlighted the dysregulation of branched-chain amino acids, tryptophan, arginine and other metabolic pathways that could contribute to inflammation, oxidative stress, endothelial dysfunction and myocardial remodeling. These data imply that the metabolic repercussions of gut dysbiosis are not limited to TMAO, but rather complicated host–microbiome interactions in cardiovascular homeostasis [65].

Differences in heart failure characteristics may potentially have a role in the variability of microbial fingerprints. HFpEF cohorts, characterized by older subjects, hypertension and metabolic comorbidities, tend to show more diet- and inflammation-associated microbial shifts, while HFrEF cohorts show more consistent profound loss of core commensal taxa and increased abundance of opportunistic pathogens. These findings show that heart failure is not associated with a homogeneous microbial signature, but rather with phenotype-dependent dysbiosis patterns. Methodological heterogeneity complicates interpretation. Studies based on 16S rRNA gene sequencing give mostly relative taxonomic abundance at the genus or phylum level, which may mask strain-level variations and functional capacity. Shotgun metagenomics is more precise but less used in heart failure populations. Furthermore, metabolomics studies are results of microbial activity rather than microbial composition per se, which may explain the discrepancies in taxonomic and metabolic findings amongst studies [66,67]. Thus, the individual bacterial taxa should not be considered as a legitimate heart failure biomarker or therapeutic target at this point but rather as members of the large functional microbial networks.

Taken together, the existing data indicate that functional changes in the gut microbiome may be a more reliable marker of heart failure than changes in specific bacterial species. Although the specific taxonomic signatures differ significantly between studies, probably due to differences in patient characteristics, diet, comorbidities, medication exposure and sequencing methodologies, the consistent depletion of SCFA-producing bacteria and enrichment of pro-inflammatory microbial functions suggest common pathophysiological pathways. Thus, future research may benefit from focusing on microbial metabolic ability and host–microbe interactions rather than trying to discover a general taxonomic hallmark of heart failure [68].

## 5. Limitations and Future Perspectives

In various cohorts of patients with heart failure, reduced short-chain fatty acid (SCFA)-producing bacteria (e.g., Faecalibacterium, Roseburia and Ruminococcus) and increased pro-inflammatory taxa (e.g., Proteobacteria, Streptococcus, and Alistipes) have been observed. These microbial changes seem to be closely related with systemic inflammation, decreased intestinal barrier function and increased levels of trimethylamine N-oxide (TMAO). Together, all of these bacteria have been repeatedly linked to worse cardiovascular outcomes and greater disease severity. In addition, a bidirectional gut–heart–brain axis has been identified, implicating that changes in the microbiota may affect autonomic activity and cardiac remodeling.

Despite these common patterns, substantial limitations persist. Most studies are cross-sectional, limiting causal inference, and there is substantial variability in research groups, dietary habits, comorbidities and microbiome investigation methodologies (16S rRNA sequencing vs. metagenomics). Metabolomic studies are restricted to TMAO and SCFAs, leaving maybe other potentially significant microbial metabolites underexplored.

Most of the studies mention the presence of noticeable microbial dysbiosis in heart failure, but there are some variations in the taxonomic changes reported. Beale et al. did not find significant changes in the Firmicutes/Bacteroidetes ratio among HFpEF patients compared to controls [29]. Zhang et al. [37], however, found a depletion of Bacillota with Bacteroidota expansion. Similar discrepancies have been reported in other sequencing studies. The differences may be due to the heterogeneity of patient characteristics, dietary habits, drug exposures, disease severity, geographical location, and the analytical pipelines used to profile the microbiome. Nevertheless, studies consistently report a decline in beneficial SCFA-producing bacteria and enrichment of potentially harmful microorganisms, indicating that functional changes might be more meaningful than changes in particular taxa despite these taxonomic disparities.

There is a clear need in the future to focus on longitudinal and interventional research to clarify the causal links between the gut microbiota and the progression of heart failure. Multi-omics methods such as metagenomics, metabolomics and transcriptomics could be integrated to identify novel biomarkers and therapeutic targets. Finally, the identification of microbiome signatures specific to particular heart failure phenotypes (e.g., HFrEF vs. HFpEF) may open the way for more tailored and mechanistically informed treatments.

## 6. Directionality of the Gut–Heart Axis

A major subject yet unanswered in gut–heart axis research is the directionality of the association between heart failure and gut dysbiosis. On initial inspection, it seems to represent the traditional “chicken-and-egg” dilemma: Does dysbiosis cause the development of heart failure, or does heart failure cause dysbiosis? However, current research suggests that both mechanisms might occur, but at different stages of illness progression.

The fundamental pathological cause in early heart failure appears to be the cardiac dysfunction itself. Reduced cardiac output, intestinal hypoperfusion, venous congestion and intermittent mucosal ischemia can compromise the integrity of the intestinal barrier and modify the local intestinal environment. These hemodynamic abnormalities contribute to microbial imbalance, reduced microbial diversity, disruption of epithelial tight junctions and increased intestinal permeability. Dysbiosis at this stage is mostly due to cardiovascular disease.

There may be a kind of causal inversion as the condition progresses. The altered microbiota has an increasingly active pathophysiological function via the production of bioactive metabolites and inflammatory mediators. Systemic inflammation, endothelial dysfunction, oxidative stress, neurohormonal activation and myocardial remodeling may be promoted by increased production of metabolites such as trimethylamine N-oxide (TMAO), altered bile acid profiles, reduced short-chain fatty acid production and translocation of bacterial products including lipopolysaccharide. In this later phase, dysbiosis may no longer be just a consequence of HF, but a metabolic and inflammatory amplifier of illness progression. This approach could be considered as a vicious cascade, in which cardiac dysfunction induces intestinal changes that lead to further microbial dysbiosis and metabolic changes, which in turn worsen cardiovascular pathology. This concept helps reconcile apparently discordant data in the literature and explains why observational studies often discover robust correlations between changes in gut microbiota and severity of heart failure without necessarily proving dysbiosis as the major starting event.

Critically, the data available do not support a simple causal hypothesis in which a single microbial change directly leads to heart failure. Instead, the existing data indicate a complicated bidirectional connection where hemodynamic abnormalities, systemic inflammation, neurohormonal activation, comorbidities, and microbial metabolites influence each other across time. Therefore, dysbiosis cannot be seen as a single pathogenic trigger but rather as a constituent of a complex network of diseases that can participate in disease progression in particular clinical scenarios.

Moreover, the relative impact of each component of this cycle likely differs according to disease stage, heart failure profile, comorbidities and environmental factors. Future longitudinal studies and interventional trials will be required to establish the stages of disease at which microbiota-targeted therapies will be most therapeutically beneficial.

## 7. Conclusions

The gut microbiota is becoming recognized as a metabolically active ecosystem that is closely associated with cardiovascular illness, particularly heart failure. Experimental and clinical data provide support for the hypothesis that hemodynamic alterations associated with heart failure, including lower intestinal perfusion, venous congestion, and mucosal ischemia, may be involved in intestinal barrier disruption and microbial dysbiosis. According to the gut–heart hypothesis, these changes may lead to the translocation of microbial products, particularly endotoxins, into the systemic circulation, which can promote inflammatory and neurohormonal activation and perhaps contribute to unfavorable cardiac remodeling. However, these pathways are largely supported by associative and preclinical evidence and no definitive causal links have been shown in humans.

However, despite these strong connections, numerous major restrictions still exist. No conclusive cause-and-effect relationship has been established between gut microbiota changes and progression of heart failure, and the present evidence is mostly drawn from observational studies, susceptible to confounding from diet, comorbidities, medication use and disease severity. Furthermore, the heterogeneity of the methods used between research and the high inter-individual variability in microbiome composition restrict the reproducibility of certain taxonomic markers. The gut microbiota has been considered as a therapeutic target in heart failure. Diet modification, prebiotics, probiotics, selective antibiotics and fecal microbiota transplantation have shown promising results in preclinical models and early phase clinical investigations. However, the evidence from large randomized controlled studies is still missing and microbiota-targeted therapies are still not indicated for routine clinical application.

Thus, there is currently no clear evidence to support the routine clinical use of microbiome-based diagnostics in heart failure. In conclusion, the gut–heart axis is a rapidly evolving area of cardiovascular research. However, for the time being, it should be regarded as a prospective research framework, rather than as a settled clinical paradigm. Further studies should focus on standardization of methodologies, longitudinal study designs, and well-powered RCTs to elucidate the causal relationships and assess if modulation of the gut microbiome can be translated into effective and evidence-based therapeutic strategies in the setting of heart failure.

## Figures and Tables

**Table 1:** Intestinal microbiota findings in individuals with heart failure.

Study	Year	Study Type	Increased Microbial Taxa	Decreased Microbial Taxa	Metabolite	Main Finding
Kuan et al. [36]	2026	Case–control study; HFrEF patients vs. healthy controls	-	-	↑ TMAO	Elevated TMAO levels were associated with impaired cardiac function and increased HFrEF severity
Zhang et al. [37]	2023	Clinical cohort	↑ Proteobacteria and PAGln-associated bacteria	↓ Beneficial Firmicutes taxa	↑ PAGln	Elevated PAGln associated with severe HF progression
Ahmad et al. [38]	2023	Observational study	Altered gut microbial metabolic signatures	-	↑ TMAO, inflammatory metabolites	Gut microbiome-derived metabolites were associated with HF severity and systemic inflammation
Sun et al. [30]	2022	Case–control	↑ Proteobacteria, ↑ pathogenic bacteria such as *Escherichia-Shigella*	↓ Firmicutes, ↓ *Faecalibacterium*	-	Dysbiosis severity correlated with advanced HF severity
Anderson et al. [28]	2022	Observational cohort	↑ TMAO-producing taxa including Enterobacteriaceae	↓ SCFA-producing taxa	↑ TMAO	Elevated TMAO linked with hospitalization and mortality
Beale et al. [29]	2021	Case–control	↑ *Enterococcus*, inflammatory-associated taxa	↓ *Ruminococcus*, ↓ SCFA-producing bacteria	-	HFpEF patients present distinct dysbiotic microbiome signatures
Israr et al. [35]	2021	Prospective cohort	-	-	↑ TMAO and gut-related metabolites	Elevated gut-derived metabolites were associated with worse prognosis and adverse outcomes in acute heart failure
Kummen M et al. [31]	2018	Case–control; HF patients vs. controls	↑ Escherichia, Shigella	↓ Butyrate-producing bacteria (e.g., *Faecalibacterium*)	↓ SCFA	Gut microbiota composition correlates with HF severity and inflammation
Luedde et al. [32]	2017	Case–control study; HFrEF patients vs. healthy controls	Altered beta-diversity and dysbiotic microbiota composition	↓ *Ruminococcaceae*, ↓ *Blautia*, ↓ *Collinsella*, ↓ core intestinal taxa	↓ SCFA-associated bacterial taxa	HF was associated with depletion of core intestinal microbiota and reduced microbial diversity
Tang et al. [39]	2014	Prospective cohort	-	-	↑ TMAO	First evidence linking TMAO to HF prognosis and mortality

Notes: ↓= decreased, ↑ = increased.

## Data Availability

No new data were created or analyzed in this study. Data sharing is not applicable to this article.

## Abbreviations

The following abbreviations are used in this manuscript:

HF	Heart failure
AHA	Acute heart failure
SCFAs	Short-chain fatty acids
LPS	Lipopolysaccharides
LVEF	Left Ventricular Ejection Fraction
NT-proBNP	N-terminal pro-B-type Natriuretic Peptide SCFAs—Short-Chain Fatty Acids
TMAO	Trimethylamine-N-oxide
HFpEF	Preserved ejection fraction
HFrEF	Reduced ejection fraction
FMT	Fecal microbiota transplantation
TAC	Transverse aortic constriction
